# Site-to-site interdomain communication may mediate different loss-of-function mechanisms in a cancer-associated NQO1 polymorphism

**DOI:** 10.1038/srep44532

**Published:** 2017-03-14

**Authors:** Encarnación Medina-Carmona, Jose L. Neira, Eduardo Salido, Julian E. Fuchs, Rogelio Palomino-Morales, David J. Timson, Angel L. Pey

**Affiliations:** 1 Department of Physical Chemistry, Faculty of Sciences, University of Granada, Av. Fuentenueva s/n, 18071, Granada, Spain; 2 Instituto de Biología Molecular y Celular, Universidad Miguel Hernández, Avda. del Ferrocarril s/n, 03202, Elche, Alicante, Spain; 3 Instituto de Biocomputación y Física de los Sistemas Complejos (BIFI), 50009, Zaragoza, Spain; 4 Hospital Universitario de Canarias, Centre for Biomedical Research on Rare Diseases (CIBERER), Tenerife, Spain; 5 Institute of General, Inorganic and Theoretical Chemistry, Faculty of Chemistry and Pharmacy, University of Innsbruck, Innsbruck, Austria; 6 Department of Biochemistry and Molecular Biology I, Faculty of Sciences, University of Granada, Av. Fuentenueva s/n, 18071, Granada, Spain; 7 School of Pharmacy and Biomolecular Sciences, The University of Brighton, Brighton, UK

## Abstract

Disease associated genetic variations often cause intracellular enzyme inactivation, dysregulation and instability. However, allosteric communication of mutational effects to distant functional sites leading to loss-of-function remains poorly understood. We characterize here interdomain site-to-site communication by which a common cancer-associated single nucleotide polymorphism (c.C609T/p.P187S) reduces the activity and stability *in vivo* of NAD(P)H:quinone oxidoreductase 1 (NQO1). NQO1 is a FAD-dependent, two-domain multifunctional stress protein acting as a Phase II enzyme, activating cancer pro-drugs and stabilizing p53 and p73α oncosuppressors. We show that p.P187S causes structural and dynamic changes communicated to functional sites far from the mutated site, affecting the FAD binding site located at the N-terminal domain (NTD) and accelerating proteasomal degradation through dynamic effects on the C-terminal domain (CTD). Structural protein:protein interaction studies reveal that the cancer-associated polymorphism does not abolish the interaction with p73α, indicating that oncosuppressor destabilization largely mirrors the low intracellular stability of p.P187S. In conclusion, we show how a single disease associated amino acid change may allosterically perturb several functional sites in an oligomeric and multidomain protein. These results have important implications for the understanding of loss-of-function genetic diseases and the identification of novel structural hot spots as targets for pharmacological intervention.

Native proteins are heterogeneous ensembles sampling a wide variety of microscopic conformations^1^. The relative population of these states is determined by their intrinsic stabilities (i.e. *free energies*) as well as their rates of interconversion (i.e. *kinetic barriers*)^1^,^2^. Large-scale and slow collective motions (in the ms to s time scales) determine the transitions between a small number of low energy states that correspond to different *conformational* states (with intrinsically different structure and energetic balance)^1^,^2^,^3^,^4^. Within these low energy states, small-scale fluctuations rapidly occur, such as backbone and side-chain dynamics, which are the main source of *conformational dynamics*^1^,^3^,^4^. A deep structural, thermodynamic and kinetic understanding of collective and local protein motions is essential to understand protein function, regulation, degradation and evolution^4^,^5^,^6^,^7^,^8^,^9^,^10^.

Protein allostery is the phenomenon that allows communication between two distant sites in a protein (e.g. two binding sites for different ligands or between a ligand binding and a mutated/post-translational modification site)^11^. Ligand binding remodels protein conformational ensembles by stabilizing binding competent states from such a conformationally heteregenous pre-existing equilibrium. The consequent population-shift of microstates is *macroscopically* observed as a *conformational change*^11^,^12^,^13^,^14^. Accordingly, structural and dynamic aspects of these conformational changes have significant impact on protein function and regulation (e.g. on ligand binding affinity and energetics)^2^,^3^,^4^,^12^,^15^. A combination of functional, structural and thermodynamic analyses is a powerful approach to identify allosteric networks and pathways in proteins and to distinguish between different allosteric mechanisms (e.g. *induced-fit* vs. *conformational selection*)^4^,^12^,^14^,^16^.

Disease-associated inherited changes in protein sequence (i.e. mutations and polymorphisms) affect protein activity, regulation and stability^17^,^18^,^19^,^20^,^21^,^22^. However, allosteric site-to-site communication underlying loss-of-function genetic variations has rarely been investigated in multi-domain oligomeric proteins^23^,^24^,^25^. Here, we hypothesize that the cancer-associated single nucleotide polymorphism p.P187S (rs1800566/c.C609T) in the NADP(H):quinone oxidoreductase 1 (NQO1; EC 1.6.5.2) mediates enzyme loss-of-function through long-range site-to-site effects originating at the p.P187S site and communicated to distant functional sites through a hypothetical *allosteric interaction network* (Fig. 1). NQO1 is a two-domain FAD-dependent enzyme (Fig. 1A,B) involved in the two-electron reduction of quinones, important for Phase II detoxification reactions, superoxide scavenging and activation of certain cancer pro-drugs^26^. The N-terminal domain (NTD) binds FAD and the smaller C-terminal domain (CTD, residues 225–274) is structurally involved in binding of NADH, substrates and competitive inhibitors (e.g. dicoumarol) (Fig. 1B,C). NQO1 also interacts with and stabilizes cancer-associated transcription factors such as p53 and p73α^27^,^28^,^29^,^30^, even though the structural basis of this intracellular interaction is unknown. Although p.P187S strongly reduces NQO1 levels and activity *in vivo*, crystallographic analyses of holo-p.P187S have shown no significant effect on functional or structural sites such as FAD binding site, the CTD or the monomer:monomer interface^31^. However, molecular dynamics (MD) simulations, biophysical experiments and expression analyses have supported the hypothesis that p.P187S inactivates and destabilizes NQO1 through dynamic changes in the apo-state, in particular on two functionally and structurally distant sites (Fig. 1D): the FAD binding site, associated with enzyme inactivation; and the CTD, associated with enhanced proteasomal degradation of p.P187S^22^,^24^,^31^.

In this work, we investigate how the p.P187S polymorphism affects the FAD binding site and the CTD of NQO1 through long-range communication of structural and dynamic perturbations (Fig. 1D), as well as their potential effects on the binding site of oncosuppresors. To do so, we characterize wild-type (WT) and p.P187S NQO1 in their full-length and C-terminal truncated (Δ50-NQO1) forms using a multi-disciplinary approach. Overall, we show how a single amino acid substitution may cause protein loss-of-function by simultaneously affecting multiple functional sites in a complex oligomeric and multidomain protein with metabolic and regulatory roles.

## Results

### The CTD of NQO1 plays an important role in the conformational equilibrium of the apo-state

**Figure 1:**
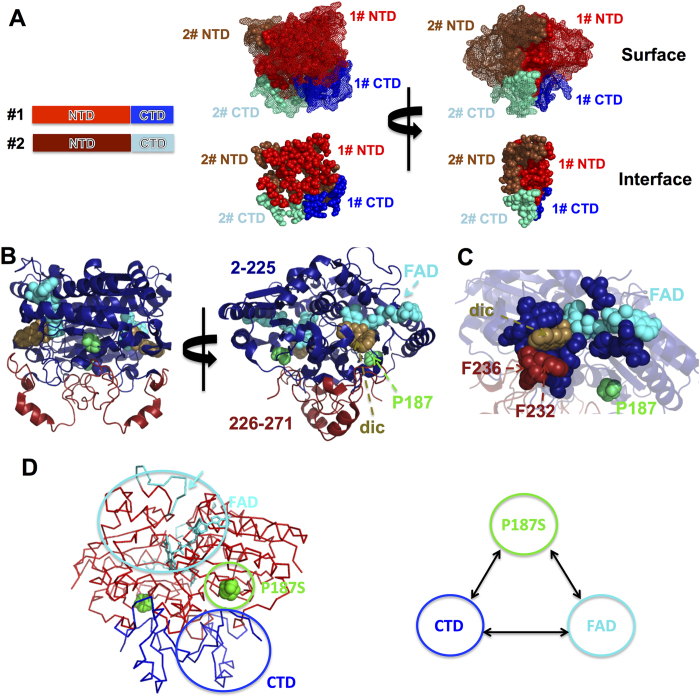
NQO1 structure, dimerization and functional definition of the hypothetical allosteric interaction network. (**A**) Two views of the NQO1 dimer showing the protein surface and the dimerization interface (as calculated using the PISA server^34^); the N-terminal domains (NTD) are displayed in red (monomer #1) and brown (monomer #2) while the C-terminal domains (CTD) are displayed in blue (monomer #1) and cyan (monomer #2); (**B**) Two views of the NQO1 dimers displaying secondary structures of NTD (residues 2-225, in blue) and the CTD (residues 226–271, in red). FAD, dicoumarol (dic) and P187 are shown in cyan, light brown and green, respectively. (**C**) Close-up view of the FAD and dicoumarol binding sites, with the residues belonging to the NTD (blue) and CTD (red) highlighted. (**D**) Schematic representation of the hypothesized allosteric interaction network involving the p.P187S and FAD binding sites and the CTD. In the FAD binding site (left panel), the highly dynamic loop 57–66 in the apo-state of p.P187S is indicated with a cyan arrow to highlight its far location from the CTD and p.P187S sites. Structures were displayed with Pymol^60^ using the NQO1 structure (PDB:2F1O).

Structural analyses^32^ have shown that the CTD forms part of NQO1 dimer interface (Fig. 1A,B), adding up to 35% of the total buried surface at this interface (960 Å^2^ of 2700 Å^2^ per monomer; calculated using cpptraj^33^). Calculations using the PISA server (http://www.ebi.ac.uk/pdbe/pisa/34) reveal that at least one residue of the CTD is involved in 40% of the hydrogen bonds and half of the salt bridges at the dimer interface. In addition, the CTD forms part of the dicoumarol binding site (Phe232 and Phe236; Fig. 1C), but it does not seem essential for FAD binding or stable folding of the NTD (Figure S1^31^). While Pro187 is not close to the FAD or dicoumarol binding sites, its shortest distance to the CTD is about 4 Å (with Gln268 and Ile269) (Fig. 1B,D). Therefore, it is unclear whether deletion of the CTD affect the dimerization, activity and stability of NQO1, as well as the structural and dynamic effects caused by p.P187S on the FAD binding site and the CTD (Fig. 1C,D and ref. 24).

NQO1 WT and p.P187S in their full-length and C-terminal deleted (lacking the last 50 residues at the C-terminus, Δ50-NQO1) versions were expressed in and purified from *E. coli*. To investigate their oligomeric state, their hydrodynamic behavior was analyzed by size-exclusion chromatography (SEC) and dynamic light scattering (DLS) (Figure S2A and S2B). Our results show that all NQO1 variants behave as dimers in solution even at concentrations ≤1 μM (note that dimers of NQO1 and Δ50-NQO1 are expected to have a molecular weight of 65.2 and 53.9 kDa, respectively). The 1-1 echo pulse sequence of NQO1 WT and P187S mutant were also used to measure *T*_2_ relaxation times of the most down-field isolated amide protons of both spectra, yielding a molecular weight of approximately 78 kDa (at 20 μM concentration in monomer units; see Supplementary information). We attempted to detect dimer dissociation and to determine their dissociation constants by isothermal titration calorimetry (ITC; Figure S2C). However, dilution of NQO1 dimer caused little or no heat change compared to the blank (i.e. buffer) dilution heats, thus supporting that dissociation of dimers in the low micromolar range initially used during titrations occurs to little or negligible extent. This result is important considering that the amount of surface exposed to the solvent upon dimer dissociation (expected to be about 5400 Å^2^; see above) would give a remarkable heat release, in the 10–15 kcal·mol^−1^ range at 25 °C, using the previously published structure-energetics correlations^35^.

**Figure 2:**
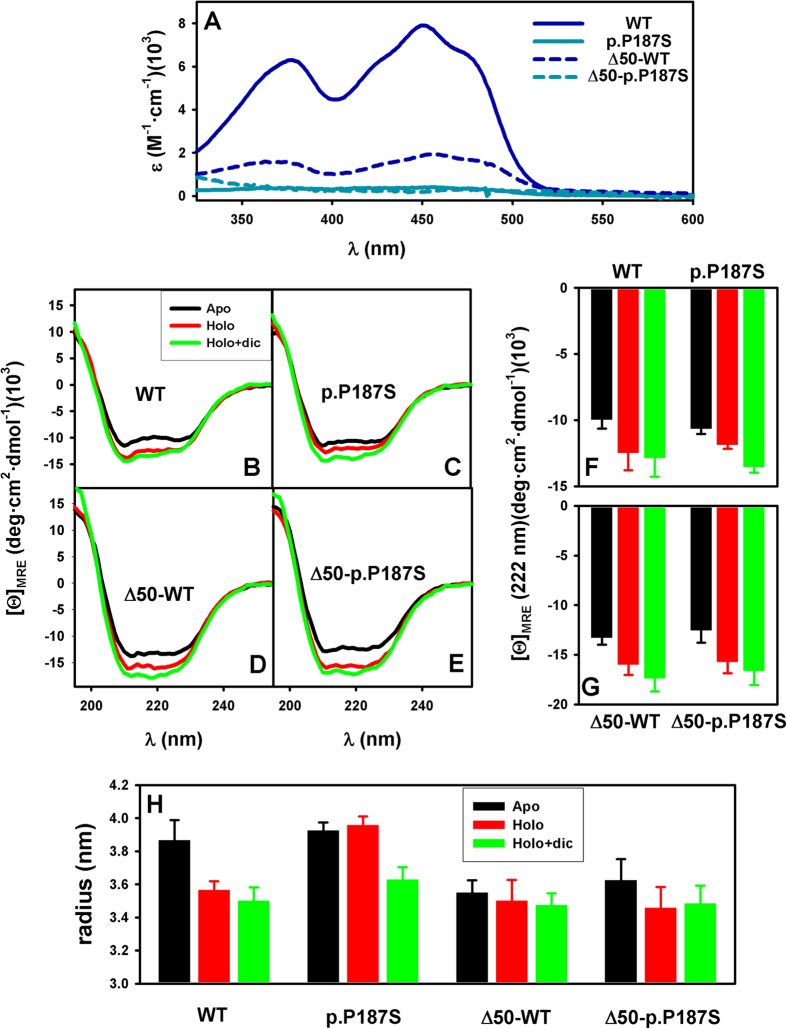
Conformational analysis of NQO1 variants. (**A**) FAD bound to the proteins as purified from Near-UV/visible absorption spectra. (**B**–**E**) Far-UV CD spectra of NQO1 variants as apo-proteins, in the presence of FAD (Holo) or FAD + dicoumarol (Holo + dic); (**F**,**G**) values of mean residue ellipticity ([Θ]_MRE_) at 222 nm from spectra shown in panels (**B**–**E**,**H**) hydrodynamic radius for NQO1 variants in the different ligation states. Data in panels A–G are the average from three independent experiments, while in panel H are from 3 to 6 independent experiments. Values in panels F, G and H are mean ± s.d. from replicates. In all cases, two to three different protein purifications were used, and the temperature was 25 °C.

Remarkably, NQO1 proteins as purified show different amounts of FAD bound, with WT having the highest content, Δ50-WT showing intermediate levels (about 4-fold lower than for WT), and p.P187S and Δ50-p.P187S displaying negligible levels of bound FAD, as judged from their corresponding absorption near-UV/visible spectra (Fig. 2A). To investigate their overall conformation, we have prepared apo-proteins and then added precise concentrations of ligands to achieve different ligation states (holo-NQO1, with FAD, and holo-NQO1+ dicoumarol, with an excess of FAD and dicoumarol). The far-UV circular dichroism (CD) spectra of full-length and Δ50 apo-proteins show a high content of ordered secondary structure (Fig. 2B–E). Binding of FAD alters the secondary structure content, increasing the ellipticity at 222 nm (a hallmark for α-helical content) by 20–25% in all cases except for p.P187S in which this increase is of 10% (Fig. 2F,G; note that in all cases, a statistical comparison of a given variants as holo- vs. apo-protein yields a p value < 0.05 using a t-test). Binding of dicoumarol, that interacts with both NTDs and CTDs in full-length NQO1, leads to further increase of 16% in holo-p.P187S (p < 0.05) but not in holo-WT, while it causes only small increments (6–9%) in Δ50 variants (Fig. 2F,G). Since apo-NQO1 intrinsically populates partially unfolded conformations^22^,^24^,^31^,^36^, these results suggest that binding of FAD and dicoumarol to full-length and Δ50 variants leads to a shift in the conformational equilibrium of NQO1 towards more compact, ligand binding competent conformations. Accordingly, DLS experiments showed a decrease in the hydrodynamic radii of WT and p.P187S when FAD or FAD + dicoumarol are added, respectively (Fig. 2H), further supporting a more compact average dimer conformation in the presence of these ligands. In the case of the Δ50 variants, binding of FAD and/or dicoumarol led to much smaller effects on the hydrodynamic radius (Fig. 2H) indicating an important role of the CTD in the conformational equilibrium of apo-NQO1.

### The dynamic CTD of p.P187S neither contributes to its low *in vitro* kinetic stability nor to ligand mediated stabilization of NQO1 dimers

**Figure 3:**
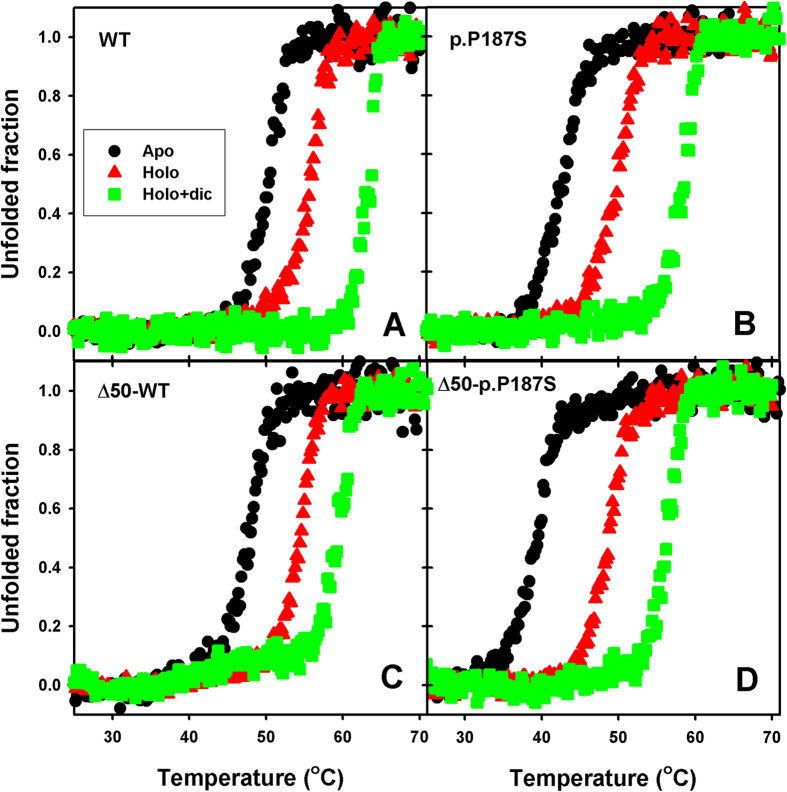
Thermal stability of NQO1 variants. Experiments were performed using NQO1 variants in different ligation states. Raw denaturation curves were normalized using linear pre- and post-transition baseline for sake of comparison.

p.P187S strongly decreases the *in vitro* thermal and kinetic stability of NQO1 by accelerating dimer unfolding and dissociation^22^,^24^. To investigate the role of the CTD on its low kinetic stability, we have performed thermal denaturation studies monitored by CD spectroscopy (Fig. 3). Removal of CTD causes a small 3 °C destabilization on the WT and p.P187S backgrounds (Fig. 3 and Table 1). A large excess of FAD (holo-proteins) causes a remarkable stabilization, ranging from 5 to 10 °C (Fig. 3 and Table 1), and an excess of FAD and dicoumarol have a roughly additive effect on stability (from 12–17 °C). These results imply that removal of CTD modestly destabilizes NQO1, and it is not the cause the low *in vitro* kinetic stability of p.P187S.

**Figure 4:**
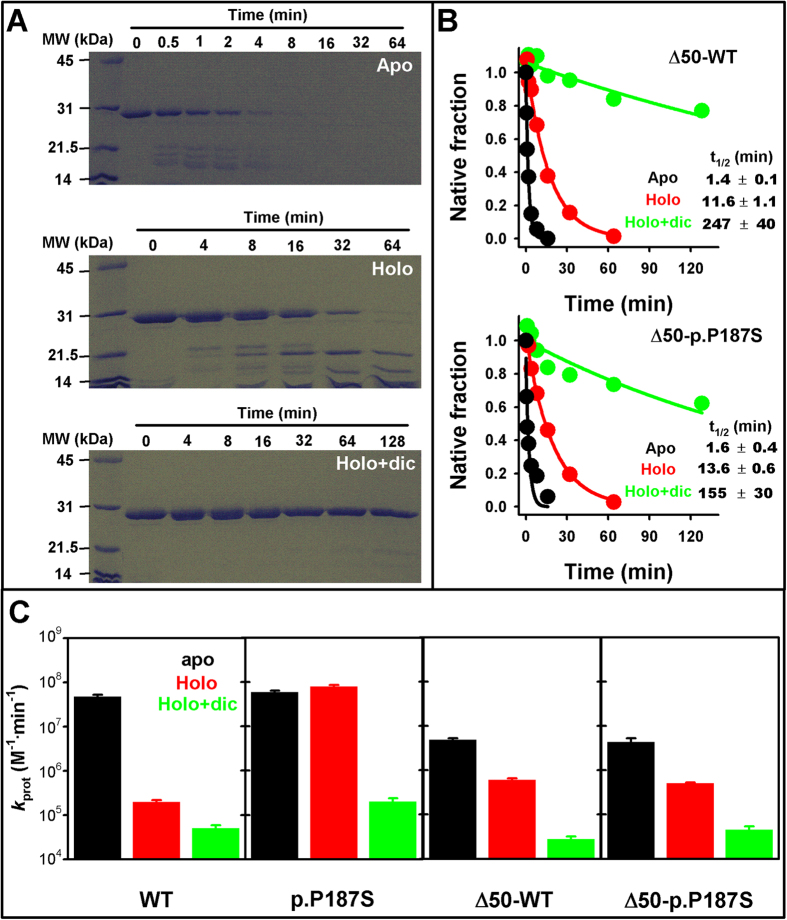
Partial proteolysis of Δ50-NQO1 variants. (**A**) representative SDS-PAGE gels of degradation kinetics of Δ50-WT in different ligation states (apo, holo and holo-holo + dic). The regions corresponding to molecular sizes from 45 to 14 kDa bands are shown for sake of clarity (see Figure S3 for additional pictures of gels); (**B**) Time-course of the degradation of the native band of Δ50 variants. Experiments performed at 25 °C in the presence of 0.1 μM of thermolysin. (**C**) proteolysis rate constants for full-length and Δ50-NQO1 variants in different ligation states (data for full-length variants are from ref. 24).

Partial proteolysis of full-length NQO1 proteins provides quantitative information on the effects of polymorphisms and ligand binding on local dynamics^24^. Partial proteolysis of Δ50 variants as apo-proteins showed a rapid decay of the native protein (Fig. 4A,B), and the appearance of 3–4 proteolysis products with molecular sizes of 16–19 kDa (Table S1) based on HPLC/ESI-MS. FAD binding causes a remarkable 10-fold increase in the resistance towards degradation (Fig. 4A,B) and the accumulation of 18 and 15 kDa partially proteolyzed species (Table S1). Binding of dicoumarol to holo-proteins leads to a 10–20 fold additional stabilization of the native Δ50 protein (Fig. 4A,B). Peptide fingerprinting of digested samples by MS/MS revealed that cleavage is initiated in the C-terminal part of Δ50-NQO1, involving several active cleavage sites in the region 150–170 (particularly dynamic in the apo-state of Δ50-NQO1, see our MD simulations below) leading to the formation of a stable intermediate comprising residues 1–143 (Figs 4A and S3). Our comparative analyses of proteolysis kinetics supports our previous proposal of a very dynamic CTD in the apo-state of NQO1, that remains dynamic in the holo-p.P187S unless dicoumarol is bound (Fig. 4C). We must note that an absolute comparison of local dynamics between NQO1 and Δ50 variants is not possible because experimental proteolysis rate constants are proportional to the sequence-dependent intrinsic proteolysis rate constants for primary cleavage sites under the conditions used.

### The CTD of p.P187S undergoes a large conformational rearrangement upon dicoumarol binding and contributes to long-range dynamic effects affecting FAD binding

**Figure 5:**
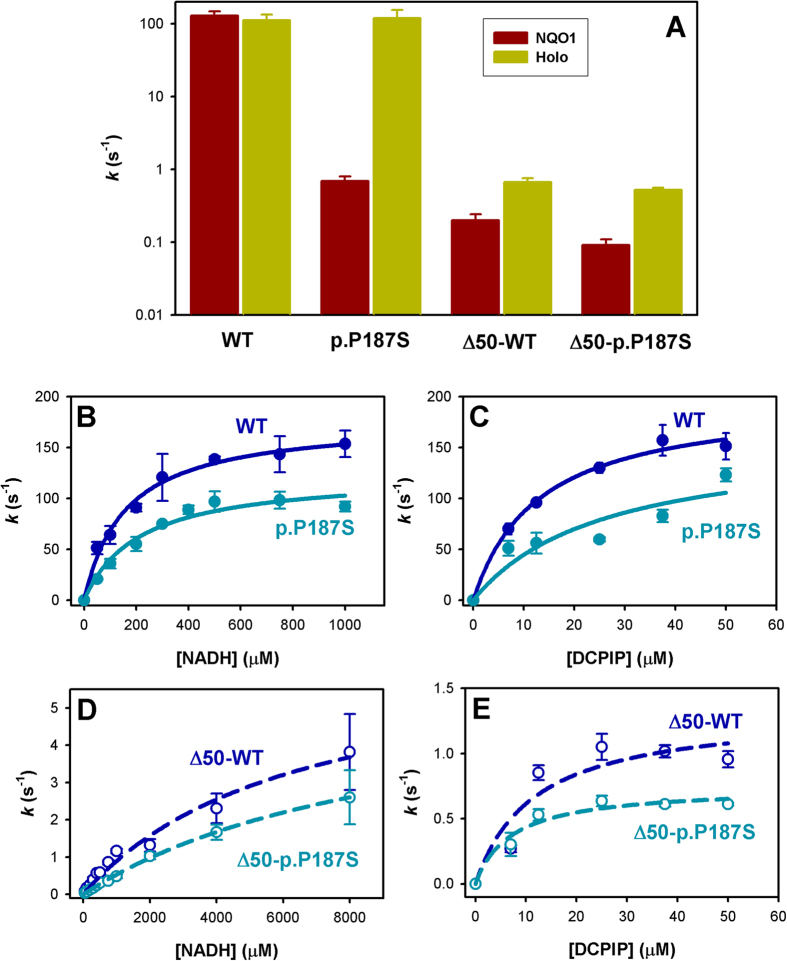
Enzyme kinetic analyses of NQO1 variants. (**A**) Activity measurements in the absence (NQO1) or the presence (Holo) of a FAD excess, using 70 μM DCPIP and 1 mM NADH. Please, note the logarithmic scale of the y-axis. (**B**) Activity dependence on NADH (**B**,**D**) and DCPIP (**C**,**E**) concentrations for WT/p.P187S (**B**,**C**) and Δ50-WT/Δ50-p.P187S (**D**,**E**). Lines are fits to the Michaelis-Menten equation.

The low intracellular specific activity of p.P187S is largely associated with defective FAD binding (Fig. 5A and ref. 24). Removal of the CTD causes a large decrease in specific activity, that arises from a reduction in catalytic performance rather than in FAD content, since saturation with FAD only causes a modest increase in activity (Fig. 5A). The enzyme kinetic parameters for NADH and the substrate DCPIP (2,6-dichlorophenolindophenol) of holo-enzymes have been determined (Fig. 5B–E), revealing little effect of p.P187S on catalytic performance, while removal of the CTD leads to a 30-to-100-fold reduction in specific activity, and importantly, to a decrease in the apparent affinity for NADH by 30-to-50-fold (Fig. 5B–E and Table 2). These results confirm that p.P187S and deletion of the CTD cause NQO1 inactivation through different mechanisms: the former by reducing FAD binding affinity and the latter by strongly decreasing catalytic performance.

**Figure 6:**
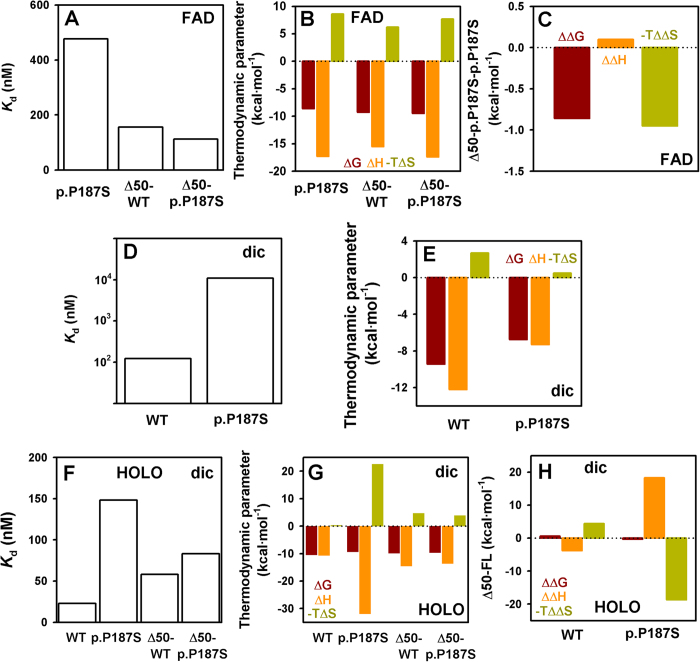
Thermodynamics of FAD and dicoumarol binding to NQO1 proteins. (**A**–**C**) Binding affinity (**A**) and thermodynamic profiles for FAD binding (**B**). In panel C, the effect of C-terminal withdrawal in thermodynamic binding parameters for p.P187S is shown. (**D**,**E**) Dicoumarol binding affinity (**D**) and thermodynamic binding profiles (**E**) for WT and p.P187S as purified. (**F**–**H**) Binding affinity (**F**) and thermodynamic profiles for dicoumarol binding (**G**) to holo-proteins. In panel H, the effects of C-terminal deletion in thermodynamic binding parameters are displayed.

At this stage, we hypothesized that the CTD may particularly affect the catalytic performance of p.P187S *in vivo* due to long-range communication with FAD binding sites (Fig. 1D). NQO1 WT displays 10–100 higher affinity for FAD than that of p.P187S and some degree of negative cooperativity^22^, making a robust comparison of their binding energetics difficult. Nevertheless, removal of the CTD abolishes the negative cooperativity of NQO1 WT (as seen by the good description of their binding isotherms using a non-cooperative binding model; Figure S4A) and also the deleterious effect of p.P187S on FAD binding, with WT and p.P187S truncated forms showing similarly high binding affinities and thermodynamic signatures (Fig. 6A,B). Notably, removal of the CTD increases the affinity of p.P187S by 4-fold due to a modest favourable entropic contribution to binding (Fig. 6C). These results reveal a contribution of the CTD to FAD binding affinity, playing opposite roles in WT and p.P187S, probably through long-range site-to-site communication between the p.P187S and *functional* CTD and FAD binding sites. This is particularly interesting because removal of CTD has little or no structural and dynamic effects on the FAD binding mode of holo-p.P187S^31^ (Figure S1 and the next section), thus indicating that communication between the p.P187S and functional sites mainly operates on the conformational equilibrium of the apo-state of p.P187S. Furthermore (see the next section), the entropic origin of the lower FAD binding affinity of p.P187S supports that it primarily arises from changes in protein conformational dynamics (i.e. *conformational entropy*, see Fig. 6C and the next section).

As purified, p.P187S binds dicoumarol with 90-fold lower affinity than NQO1 WT (Fig. 6D). This is mostly due to a much lower favourable enthalpic contribution, largely compensated by a more favourable entropic change (Fig. 6E). This large difference in affinity is likely explained by the much lower FAD content of p.P187S as purified. According to earlier crystallographic analyses and MD simulations^24^,^32^, dicoumarol binding must be favoured by bound FAD (i.e. in the holo- vs. the apo-state), primarily through stronger (enthalpy-driven) interactions that would be counterbalanced by a large decrease in protein dynamics (i.e. loss of conformational entropy). Consistently, binding of dicoumarol to the holo-enzymes (i.e. saturated with FAD) shows 5-fold (WT) and 75-fold (p.P187S) higher affinities than those found for the proteins as purified (Figs 6F and S4B). These results show the important structural and energetic roles of FAD in the efficient binding of dicoumarol, and also indicate differences in dicoumarol binding to the holo-forms of p.P187S and WT. Indeed, binding of dicoumarol to holo-p.P187S shows a much larger favourable enthalpic contribution (about −21 kcal·mol^−1^) which is compensated by a large decrease in entropy (Fig. 6G). A plausible explanation for this could be a large loss of solvation/conformational entropy counterbalanced by the structural reorganization of the protein, particularly at the highly dynamic CTD of p.P187S. In fact, simple structure-energetic correlations for protein folding^35^ provides an enthalpy change at 25 °C of ∼0.21 kcal·mol^−1^·(residue)^−1^, consistent with a much larger conformational rearrangement (roughly a hundred residues) in p.P187S upon dicoumarol binding. According to this, dicoumarol binding to holo-Δ50-WT and holo-Δ50-p.P187S is very similar (Figure S4C), confirming that dicoumarol binding to the full-length holo-p.P187S is associated with large structural and dynamic rearrangements at its CTD (Fig. 6G,H).

### Molecular dynamics (MD) simulations support the role of the CTD in the communication between p.P187S and FAD binding sites

**Figure 7:**
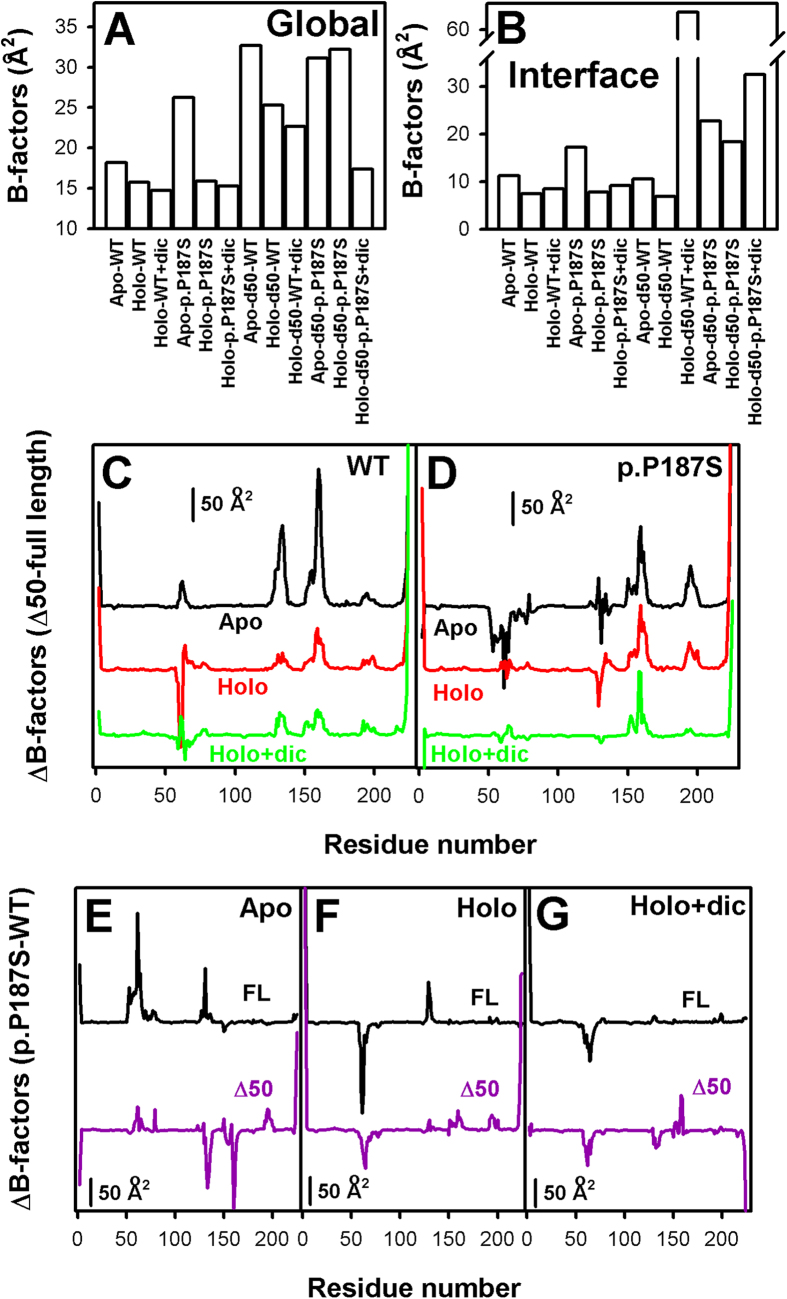
Dynamic effects (B-factors) of CTD truncation of NQO1 and ligand binding investigated by MD simulations. (**A**,**B**) Effects on the global (**A**) and dimer interface (**B**) dynamics of full-length and truncated NQO1 variants in different ligation states (apo, holo, and holo + dicoumarol); (**C**,**D**) Difference between C-terminal truncated and full length dynamics for WT (**C**) and p.P187S (**D**) in different ligation states; (**E**–**G**) Difference between p.P187S and WT as full-length and C-terminal truncated proteins in different ligation states ((**E**), apo; (**F)**, holo; (**G)**, holo + dicoumarol).

Molecular dynamics (MD) simulations have shown that p.P187S affects fast internal protein dynamics through long-range effects affecting the FAD binding site and the CTD^24^. To investigate whether CTD removal could intefere with the dynamic communication between p.P187S and the FAD binding sites, we performed MD simulations on Δ50-NQO1 variants, and analyzed their backbone dynamics using two relevant parameters^24^: B-factors (Fig. 7) and dihedral entropies (Figure S5).

Removal of the CTD increases the average flexibility of NQO1, particularly at the dimer interface, consistent with its modest *in vitro* kinetic destabilization, while thermal stabilization upon FAD and dicoumarol binding correlates well with ligand mediated reduction of global dynamics (Figs 7A,B and S5A-B). Interestingly, the decreased binding affinity for FAD in Δ50-NQO1 WT seems to originate from larger conformational fluctuations in the FAD binding site of the apo-state (at residues 57–66, 125–136 and 150–166; Figs 7C and S5C). Importantly, we observe that the CTD is essential for p.P187S to enhance local dynamics in the FAD binding site of the apo-state, particularly at the 57–66 loop (Fig. 7D). Therefore, our MD simulations explain at atomic resolution how removal of the CTD significantly enhances the binding affinity of p.P187S by a modest reduction of binding (conformational) entropy. Therefore, the CTD has opposite effects on the FAD binding site dynamics of WT and p.P187S in the apo-state (Fig. 7C,D), in agreement with our experimental observations (ref. 22 and Fig. 6A–C). Accordingly, CTD truncated forms of WT and p.P187S show similar FAD binding energetic and dynamic signatures (Figs 6B, 7E and S5E).

Regarding dicoumarol binding, holo-p.P187S binds the inhibitor with two-fold lower affinity than the truncated version due to a large entropic penalty counterbalanced by a large favorable enthalpic contribution (Fig. 6F–H). This can be explained *via* a large conformational restriction (i.e. a decrease in conformational entropy) of the region 231–236 upon binding (that includes Phe232 and Phe236, which directly interact with dicoumarol; Figs 1C, 7G, S5G and ref. 24). Moreover, this large conformational restriction, in turn, is enthalpically compensated by the additional interactions established in the dicoumarol binding site at the CTD of p.P187S and the conformational change triggered upon binding.

### Structural analyses of the interaction of NQO1 variants with the SAM domain of p73α show that an active NQO1 is not required for binding

Previous biochemical experiments have supported a role for the CTD in the interaction of NQO1 with oncosuppressors such as p73α. These interactions seem to be strengthened in the presence of NADH bound to NQO1 (even though this state is expected to be unstable, undergoing FAD reduction to FADH_2_ and subsequent NAD^+^ release), and may be inhibited by dicoumarol^30^ but not by the mechanism-based inhibitor ES936 which also interacts with the CTD^32^. Truncation of the CTD (residues 209–274) or the presence of the intracellularly unstable p.P187S also seem to weaken these protein:protein interactions^29^,^37^.

**Figure 8:**
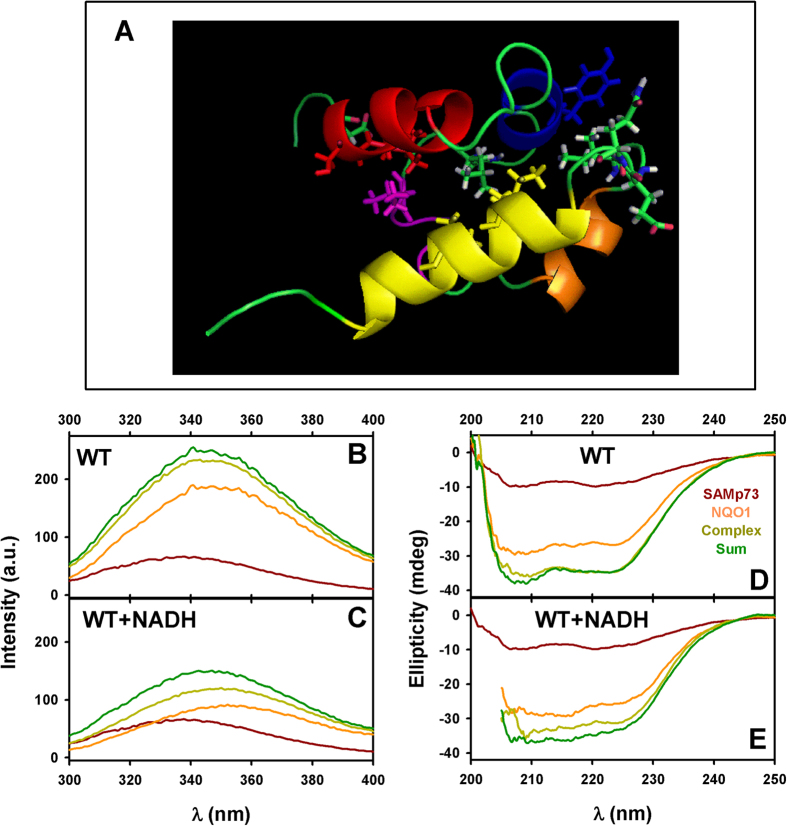
Structural analysis of the interaction between NQO1 and SAMp73. (**A**) Structure of SAMp73 (PDB: 1COK) showing the residues (in sticks) which were affected (either by signal broadening or by a CSP ≥ 0.01 ppm) (BRMB number 4413) by the presence of NQO1 WT as purified. The α-helices of SAMp73 are displayed with different colours (red for the first one; blue for the second; purple for the short 3_10_-helix; gold for the fourth α-helix; and yellow for the fifth). The figure was produced with Pymol^60^. (**B**,**C**) Fluorescence spectra (upon excitation at 280 nm) of 2 μM SAMp73 or NQO1 WT, their sum, and the spectra of an equimolar mixture (2 μM of each protein in monomer units). (**D**,**E**) Far-UV CD spectra of 10 μM SAMp73 or NQO1 WT, their sum, and the spectra of an equimolar mixture (10 μM of each protein in monomer units). In (**B**–**E**), spectra in the absence or the presence of NADH are shown in different panels.

In addition to the transactivation domain, p73 also contains a sequence-specific DNA binding domain and an oligomerization region. Particularly, the p73α splicing variant has an extended C-terminus containing a sterile alpha motif (SAMp73) capable of repressing the function of p73 transactivation domain^38^, and seemingly involved in the interaction with NQO1^30^. Therefore, we carried out NMR (HSQC) measurements on the interactions between NQO1 and SAMp73 using purified proteins. In the presence of NQO1 WT, the HSQC spectra of SAMp73 showed changes in the broadening or the chemical-shifts of the cross-peaks of a set of residues which are spatially close (Table S2), particularly in amino acids around Gly513 of SAMp73 and those involved in the N-terminal helix (Fig. 8A). Addition of NADH did not alter the set of residues affected by the presence of NQO1 WT, indicating that this interaction is not strictly NADH-dependent. Using fluorescence and far-UV CD spectroscopies (Fig. 8B–E), we also observed modest but reproducible structural changes upon NQO1:SAMp73 binding, and addition of NADH seemed to increase the extent of these structural changes. Attempts to provide quantitative data on the affinity for the interaction between NQO1 and SAMp73 by fluorescence and ITC were experimentally challenging, due to the apparent low affinity (possibly in the high micromolar range) and unstable calorimetric baselines (Figure S6 and data not shown). Since HSQC experiments showed that the spectrum of SAMp73 was not largely varied upon addition of NQO1, the changes observed are likely to occur in the environment of aromatic residues (Trp and Tyr) and the secondary structure of NQO1. Regarding SAMp73, Gly513 showed the largest changes in CSP (0.03) (Figure S7), while most of the affected cross-peaks showed broadening changes suggesting intermedium-slow kinetics for complex formation (within the NMR time scale), consistent with a low affinity interaction. Although the changes in chemical shifts observed were small, similar variations in chemical shifts have been observed in other systems^39^. These results support that the interaction between NQO1 WT and SAMp73 is not strictly dependent on the presence of NADH, but instead, NADH binding and reduction of FAD might facilitate complex formation.

The interaction of p.P187S and Δ50-WT with SAMp73 caused similar broadening in a set of signals to those found for NQO1 WT (Table S2). Δ50-WT in the presence of NADH induced broadening of all cross-peaks, and even those of Tyr487 (Ile541), Asp490, Asn504 (Met539), Gly513, Ser516, Glu535, Leu545 and Gly551 disappeared completely (there is signal overlapping between Tyr487 and Ile541, and Asn504 and Met539). Thus, removal of the CTD, the presence of p.P187S or the absence of FAD bound (note that p.P187S is essentially an apo-protein as purified) do not abolish the interaction with SAMp73 but somewhat change the interaction mode. Interestingly, the binding mode of SAMp73 to NQO1 WT is also affected by the presence of dicoumarol, showing only small CSPs in a very reduced set of signals (Leu493, Gly513 and Trp542)(data not shown).

Overall, these biophysical analyses support that while different factors affecting the activity, structure and dynamics of NQO1 (including p.P187S, cofactors and inhibitors, or the CTD) may somewhat change the binding mode to SAMp73, this interaction is not prevented by any of the aforementioned factors. Moreover, our results also provide the first structural insights into the interaction between p73α and NQO1.

### The CTD largely determines fast proteasomal degradation of p.P187S

**Figure 9:**
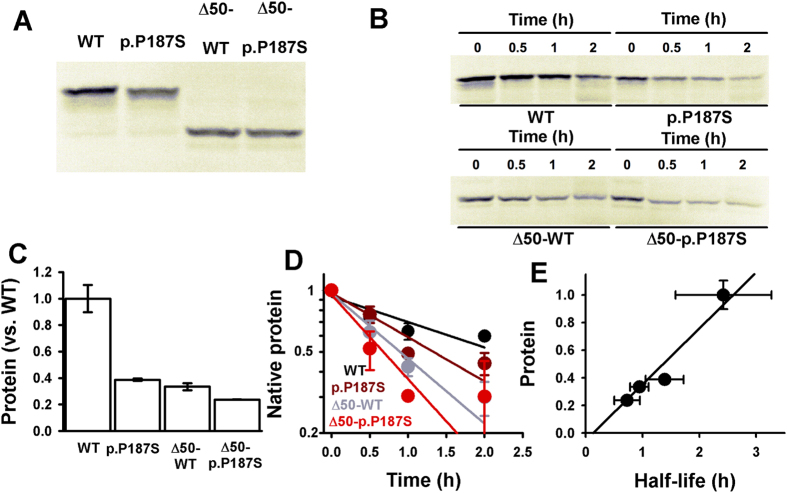
Pulse-chase experiments in rabbit reticulocytes. (**A**) Synthesis pulses of NQO1 variants. (**B**) Chase experiments. (**C**) Protein levels after synthesis pulses (normalized vs. NQO1 WT levels); (**D**) Semilogarithmic plots for chase kinetics; (**E**) correlation between protein levels after synthesis pulses and degradation half-lives. Data are mean ± s.d. from three independent experiments.

The highly dynamic CTD of p.P187S appears to act as an efficient initiation site for its proteasomal degradation^24^. Consistently, removal of the CTD had significant effects on protein levels and degradation rates upon expression in an eukaryotic expression cell free system (Fig. 9). In pulse synthesis, p.P187S showed 2.4-fold lower protein levels compared with WT as full-length proteins, while removal of the CTD (i.e. in Δ50 variants) caused a much larger decrease in WT protein levels than in p.P187S (3-fold vs. 1.5-fold; Fig. 9A–C). From chase experiments, we also observed a strong correlation between protein levels and degradation rates after a pulse of synthesis, supporting that proteasomal degradation rates strongly determine protein steady-state levels (Fig. 9B–E). Accordingly, p.P187S was degraded 1.75-fold faster than NQO1 WT (Fig. 9B,D), and removal of the CTD accelerated degradation of WT by 2.5-fold, but only that of p.P187S by 1.3-fold (Fig. 9B,D). These results show that removal of the CTD destabilizes NQO1 WT to a larger extent than the polymorphism, and therefore, the highly flexible CTD of p.P187S accelerates NQO1 proteasomal degradation. The enhanced degradation of Δ50-NQO1 is likely to be associated to its higher flexibility compared to that of full-length enzyme, as supported by our MD simulations (Fig. 7).

## Discussion

**Figure 10:**
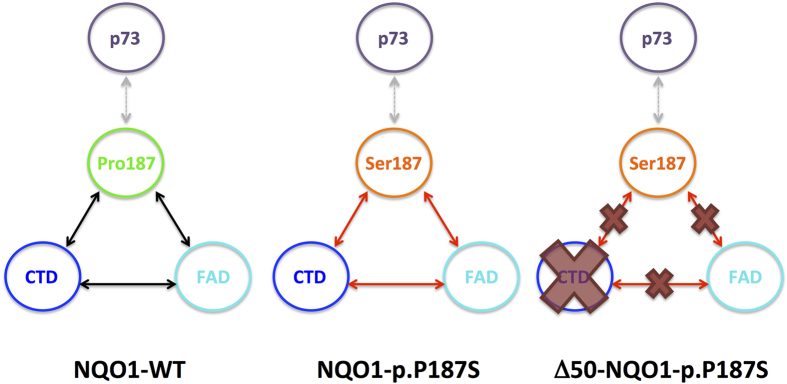
Schematic representation of the long-range communication between functional sites in NQO1 WT and p.P187S (*allosteric interaction network*). In NQO1 WT, the allosteric network between the P187 site, FAD binding site and CTD may contribute to the high-affinity cooperative binding of FAD and consequent structural and dynamic effects on the CTD upon binding. In NQO1 p.P187S, the S187 site affects FAD binding by changing the dynamics of its binding site and preventing structural and dynamic changes on CTD upon FAD binding. Removal of the CTD abolishes the dynamic effects of the S187 site on the FAD binding site. In all cases, alterations in this allosteric network seem to have a weak effect on the interaction site with p73α.

In this work, we show that the CTD plays key functional and regulatory roles in NQO1, and remarkably, how it is crucial for the manifestation of the multiple deleterious effects of the cancer-associated p.P187S polymorphism. As expected from previous crystallographic evidence, the CTD is important for NQO1 activity, providing proper orientation of the coenzymes (FAD and NADH) and the substrate. Unexpectedly, we provide evidence for the existence of long-range communication between the p.P187S site and functional sites such as the CTD and the FAD binding sites, that we therefore refer to as the *allosteric interaction network* (Fig. 10). For NQO1 WT, we propose that this allosteric network contributes to its very high affinity for FAD as well as binding negative cooperativity, which are largely perturbed upon removal of the CTD. In addition, p.P187S may disturb this allosteric network promoting dynamic and structural alterations in the FAD binding site and the CTD and consequently reducing the binding affinity for FAD and dicoumarol. Perturbed allosteric communication between FAD binding sites and the CTD is further supported by the strong effect of CTD withdrawal in p.P187S, that eliminates the effects of the polymorphism on FAD binding site dynamics of the apo-state thus increasing its FAD binding affinity. Interestingly, other functional sites (such as the binding site for p73α) may not be strongly connected to this allosteric interaction network involving the FAD binding site, the CTD and the p.P187S site (Fig. 10).

FAD and dicoumarol cause different conformational changes upon binding to WT and p.P187S. FAD binding to NQO1 WT causes larger changes in hydrodynamic volume, secondary structure and intrinsic dynamics than those in p.P187S, while dicoumarol binding has larger effects on the conformation of holo-p.P187S (Figs 2,6 and 7, and ref. 24). These different conformational changes are mostly associated with the more dynamic and less structured CTD in the holo-state of p.P187S. Accordingly, structural calculations applied to dicoumarol binding energetics supported a much larger conformational change in the CTD for p.P187S (energetically resembling *folding* of a small protein). This scenario is consistent with a pre-existing conformational equilibrium in which apo-NQO1 mainly populates states non-competent for FAD binding, and that the presence of FAD shifts the conformational equilibrium towards less dynamic and more structured FAD binding competent states (this population-shift is macroscopically observed as a *conformational change*). For dicoumarol binding, the conformational change caused in p.P187S is larger than that of WT, due to the flexible and partially unstructured CTD of the polymorphism in the unbound holo-state, that must undergo large changes in structure and dynamics to reach the binding competent state (thus penalizing its binding). Importantly, we also provide evidence that p.P187S affects locally functional dynamics of the unbound conformational ensembles, particularly at the FAD binding of the apo-state (relevant for FAD interaction) and the CTD of the holo-state (important for dicoumarol binding). Unfortunately, the available data do not allow distinguish between *induced-fit* or *conformational-selection* mechanisms for FAD and dicoumarol binding to NQO1, that distinction would depend on whether binding competent states are significantly populated in the conformational equilibrium of apo-NQO1^12^.

Proteasomal degradation of proteins is central to understanding the effect of genetic variations in loss-of-function inherited diseases^20^. In this context, dynamic or unstructured regions are key to drive recognition and efficient degradation by 20S and 26S proteasomes^8^,^9^. NQO1 is an excellent model to investigate proteasomal protein degradation, with particularly interesting roles of genetic variations, ligand binding and protein stability and dynamics in the modulation of degradation rates^8^,^22^,^24^,^36^,^40^. In the present work, we show that the enhanced dynamics of the CTD is key to determining the proteasomal degradation of NQO1, and furthermore, that once the CTD is removed (for instance by proteasomal degradation of this domain), the remaining protein is much more flexible and efficiently degraded (Figs 7 and 9). This result supports a step-wise directional degradation of NQO1 through its C-terminal end. More generally, our work highlights the idea of investigating mutational effects on protein dynamics as well as on global stability to assess the role of enhanced proteasomal degradation in loss-of-function genetic diseases.

To further understand the roles of NQO1 in human physiology and pathology, we must know the molecular and structural basis of its ability to interact with cell cycle regulators such as p53 and p73α and its inhibitory effect on the 20S proteasome^26^,^36^. Here, we present novel structural insights on the weak NQO1:oncosuppressor interaction. Importantly, we show that neither the CTD nor an active NQO1 is strictly required to interact with p73α, even though different ligands bound to NQO1 may modulate the intrinsically low affinity of this interaction, consistent with previous biochemical studies on these protein:protein interactions^29^,^30^,^37^,^41^. Interestingly enough, our studies show that the region of SAMp73 involved in binding to NQO1 is the same implicated in binding to other molecules at SAM domains: the so-called middle-loop-end-helix polypeptide-patch, with the fifth α-helix and the short 3_10_ helix as critical elements of secondary structure^42^,^43^,^44^,^45^. We must finally note that the available evidences support that intracellular destabilization of these oncosuppressors by p.P187S just mirrors its own intracellular instability rather than its inability to engage in these protein:protein interactions^24^,^29^,^36^,^40^.

In conclusion, the present work lends support to the existence of complex allosteric communication between functional sites in a two-domain oligomeric protein with a remarkable functional chemistry and how a disease-associated single amino acid change may affect different functional sites through perturbation of allosteric networks. Due to the high prevalence of missense mutations affecting protein stability, activity and regulation^19^,^21^,^46^,^47^,^48^, our mutational strategy combined with functional, structural, energetic and dynamic studies could help to decipher complex disease-associated mutational effects in many other loss-of-function genetic diseases and to identify structural targets for therapeutic correction.

## Materials and Methods

### Protein expression and purification

The introduction of full-length NQO1 variants into the pET46 Ek/LIC vector have been recently described^22^. Site-directed mutagenesis was carried out using the QuikChange kit (Stratagene) to introduce a codon stop at the 225^th^ residue of the coding sequence of NQO1 and to produce Δ50-NQO1 variants. All coding sequences were verified by DNA sequencing. Competent *E. coli* BL21 (DE3) cells were transformed with these plasmids and used to express NQO1 and Δ50-NQO1 proteins following previously described procedures^22^. Samples were buffer exchanged to 50 mM HEPES-KOH pH 7.4 and stored at −80 °C after flash-freezing in liquid nitrogen. Protein concentrations were measured spectrophotometrically using ε_280nm_ = 47900 M^−1^·cm^−1^. Correction for pre-bound FAD and preparation of apo-proteins were carried out as described^22^. NQO1 samples in different ligation states are designated as follows: apo-NQO1 (no ligand bound), NQO1 (as purified, partially saturated with FAD^22^), holo-NQO1 (saturated with FAD) and holo-NQO1 + dic (saturated with FAD and dicoumarol). FAD and dicoumarol stock solutions were prepared as previously described^22^,^24^.

SAMp73 was produced and purified as described^49^. For ^15^N-SAMp73 expression, cells were grown in M9 minimal media, with 1 gr per liter of ^15^NH_4_Cl, supplemented with vitamins, and purified as the unlabelled protein^49^.

### Isothermal titration calorimetry (ITC)

Experiments were performed in a ITC_200_ microcalorimeter (Malvern) with a cell volume of 0.205 mL. Titrations were performed in 50 mM HEPES–KOH pH 7.4. FAD titrations were carried out using 3.5–5 μM Δ50-apo-NQO1 variants (in dimer units) in the cell and 125 μM FAD in the titration syringe by performing 20–25 injections of 1.5 μL each 180 s. Dicoumarol titrations were carried out using 5 μM NQO1 or Δ50-NQO1 holo-variants (in monomer) supplemented with FAD (10 μM), and 100 μM dicoumarol + 10 μM FAD in the syringe by performing 20–25 injections of 1.5 μL each 180 s. Data were corrected for heats of dilution by performing titrations into buffer. After peak integration, binding isotherms were fitted using a model for identical and independent type of sites using the software provided by the manufacturer. Thermodynamic parameters are presented as the mean from two independent titrations.

### Far-UV circular dichroism (CD) spectroscopy

Circular dichroism (CD) was measured in Jasco J-710 or J-815 spectropolarimeters thermostatized using a Peltier element. NQO1 CD spectra were determined in 50 mM K-phosphate pH 7.4 at 25 °C, typically in a 195–260 nm range and using 50–100 nm/min scan rates. Samples of NQO1 proteins were prepared at 4 μM concentration in monomer, and 6 scans were acquired and averaged. In some cases, 10 μM FAD and 10 μM FAD + 10 μM dicoumarol were added. Experiments in the presence of SAMp73 and WT NQO1 were carried out at a equimolar concentration (10 μM in protein monomer) of each protein at 25 °C in 25 mM phosphate buffer pH 6.9. In all cases, appropriate blanks in the absence of proteins were acquired and subtracted from those of protein samples.

For thermal denaturation studies, NQO1 samples (4 μM in protein monomer) were equilibrated for 10 min at 20 °C in the absence or presence of ligands (50 μM FAD and 50 μM FAD + 50 μM dicoumarol) and thermal scans were performed up to 70–80 °C at a 2 °C/min scan rate. For sake of comparison, denaturation curves were normalized using pre- and post-transition baselines, and the half-denaturation temperature (*T*_m_) was determined as the temperature at which half of the native signal was lost.

### Dynamic light scattering

Dynamic light scattering (DLS) was carried out in a DynaPro MSX instrument (Wyatt) using 1.5 mm path length cuvettes and 10 μM protein (in monomer units) in 50 mM HEPES-KOH pH 7.4 at 25 °C. 30 measurements were acquired for each NQO1 protein, in the absence or presence of ligands (20 μM FAD and 20 μM FAD + 20 μM dicoumarol) in three to six independent replicates, averaged and used to determine the hydrodynamic radius and polydispersity using the average autocorrelation function and assuming a spherical shape.

### Fluorescence experiments

Fluorescence spectra were acquired in a Varian Cary Eclipse spectrofluorimeter (Agilent, USA) interfaced with a Peltier unit. The excitation wavelengths were either 280 or 295 nm. The emission fluorescence was collected between 300–400 nm. Excitation and emission slits were 5 nm. Spectra were corrected by subtracting the corresponding blanks. Experiments were acquired with 2 μM SAMp73 or NQO1-WT (in protein monomer), or a equimolar mixture of them, in 25 mM phosphate buffer pH 6.9 at 25 °C. NADH was added at a final concentration of 1 mM.

For titration experiments, a concentration of 4.2 μM NQO1-WT (in monomer) was used. Increasing concentrations of SAMp73 (from 0 to 8 μM) were added. The rest of the experimental set was the same as above.

### Activity measurements

NQO1 activity was measured in 50 mM HEPES-KOH pH 7.4. A reaction mixture containing recombinant NQO1 and 0.5 mM NADH was incubated at 30 °C for 5 min in 1-cm path length quartz cuvettes in a thermostatized Agilent 8453 diode array spectrophotometer. The reaction was triggered by the addition of 70 μM DCPIP (2,6-dichlorophenolindophenol) as the electron acceptor. Initial reaction rates were determined from changes in A_600nm_ resulting from the reduction of DCPIP and corrected for the non-enzymatic reaction. To determine the kinetic parameters, NQO1 enzymes were incubated with a FAD excess (1 μM final concentration), NADH was kept constant at 1 mM and DCPIP was varied from 7–50 μM or, DCPIP was mantained at 50 μM and NADH was varied from 0.05–8 mM. Blanks in the absence of proteins were determined and subtracted from the reaction with NQO1. The NQO1 concentration used varied depending on the variant to ensure linearity over time and protein concentration: 1 nM (WT with and without added FAD, p.P187S with FAD), 25–50 nM (p.P187S without added FAD), 200–600 nM (Δ50-WT and Δ50-p.P187S, with and without added FAD). The specific activity was calculated using a ε_600nm_ = 21000 M^−1^·cm^−1^ for DCPIP. *k*_cat_ and *K*_M_ values were determined using the Michaelis-Menten equation.

### Proteolysis by thermolysin

Thermolysin from *Bacillus thermoproteolyticus rokko* was purchased from Sigma-Aldrich, disolved in 50 mM HEPES-KOH 10 mM CaCl_2_ pH 7.4, stored at −80 °C and its concentration measured spectrophotometrically using a ε_280nm_ = 66086 M^−1^·cm^−1^. Proteolysis was performed at 25 °C in 50 mM HEPES-KOH 10 mM CaCl_2_ pH 7.4 using 20 μM Δ50-NQO1 (in monomer units), and the reaction was initiated by the addition of thermolysin at a final concentration of 0.1 μM. Proteolysis reactions were performed using Δ50-NQO1 as apo-proteins, upon addition of 100 μM FAD or 100 μM FAD and dicoumarol. Samples were withdrawn at different times, quenched with EDTA (20 mM final concentration) and denatured using Laemmli´s buffer at 95 °C for 5 min. Samples were resolved in 12% acrylamide SDS-PAGE gels, stained with Coomassie Blue and densitometered using ImageJ (http://rsbweb.nih.gov/ij/). After normalization of the intensities using that of the sample lacking thermolysin, the decay of the native band was analyzed using a single exponential function that yields the proteolysis rate constant *k*.

### Mass spectrometry

To analyze the proteolysis products of Δ50-WT, the apo-protein (10 μM in monomer units) was incubated at 25 °C in 50 mM HEPES-KOH 10 mM CaCl_2_ pH 7.4 with 100 nM thermolysin at three different conditions: (i) no ligand and 1 min reaction; (ii) FAD 100 μM, 16 min; (iii) FAD and dicoumarol (100 μM each) for 4 hours. The reaction volume was 150 μL. The reaction was stopped by addition of EDTA 20 mM.

125 μL of the final solution were exchanged to water using VIVAspin 500 filters (10 kDa) by two dilution-concentration cycles and submitted for High performance liquid chromatography/electrospray ionization mass spectrometry (HPLC/ESI-MS) to the High-resolution mass spectrometry unit, Centro de Instrumentación Científica (University of Granada). HPLC/ESI-MS was performed in a Acquity UPLC system (Waters), using a water/formic acid, acetonitrile/formic acid (each at 0.1%) gradient in a Acquity UPLC® BEH300 C4 column (2.1 × 50 mm, Waters) coupled to a Q-TOF Synapt62 HDMS (Waters).

10 μL of the final solution were mixed with an equal volume of Laemmli's buffer (x2), denatured at 95 °C and loaded into a SDS-PAGE gel (12% acrylamide). Two bands (#1 and #2) were manually excised from gels and submitted for analyses to the Proteomics Unit, Complutense University of Madrid, a member of ProteoRed network. Samples were in-gel reduced, alkylated and digested with trypsin, and after digestion, 1 μl of the supernatant was spotted onto a MALDI target plate, allowed to air-dry, and mixed with α-cyano-4-hydroxy-cinnamic acid matrix (Sigma) in 50% acetonitrile, and allowed again to air-dry at room temperature. MALDI-TOF MS analyses were performed in a 4800 Plus Proteomics Analyzer MALDI-TOF/TOF mass spectrometer (Applied Biosystems, MDS Sciex, Toronto, Canada). The MALDI-TOF/TOF operated in positive reflector mode with an accelerating voltage of 20000 V. All mass spectra were calibrated internally using peptides from the auto digestion of trypsin. Peptides of interest were subjected to MS/MS sequencing analyses using the 4800 plus Proteomics Analyzer. From MS spectra, suitable precursors were selected to be fragmented by CID (Collision Induced Disociation with atmospheric gas) using a 1 KV ion reflector operate method and a precursor mass window of ±4 Da. The Swissprot database (http://www.uniprot.org/help/uniprotkb) was used for protein identification without taxonomy restriction, and a home-made database with the sequence of recombinant Δ50-WT NQO1 was searched using MASCOT 2.3 (www.matrixscience.com) through the Global Protein Server v 3.6 from ABSciex, using the following parameters: enzyme, semitrypsin; carbamidomethyl cystein as fixed modification and oxidized methionine as variable modification; peptide mass tolerance, 50 ppm (PMFSearch) – 100 ppm (Combined search); up to 2 missed trypsin cleavage site; MS-MS fragments tolerance, 0.3 Da. The parameters for the combined search (Peptide mass fingerprint plus MS-MS spectra) were the same as described above. In any protein identification, the probability scores were greater than the score fixed by Mascot as significant with a p value < 0.05.

### Molecular dynamics simulations

We performed molecular dynamics simulations of Δ50-NQO1 WT and p.P187S in the apo-state and in complex with FAD and/or dicoumarol. The simulations of full-length NQO1 used as comparison are described in ref. 24. Systems were prepared in dimeric state on basis of crystal structures of the complex with FAD (PDB: 1D4A^50^) and the ternary complex (PDB: 2F1O^32^). Proteins were protonated for simulation using protonate3d and mutated as well as truncated in MOE^51^.

Simulations were performed using the GPU implementation of pmemd in Amber12^52^. Protein residues were parametrized using the Amber forcefield 99SB-ILDN^53^. FAD and dicumarol parameters were taken from an earlier study^24^. After employing an extensive equilibration protocol^54^, unrestrained systems were sampled for 100 ns in NpT ensemble and 5,000 equal-spaced snapshots were saved to trajectory.

After ensuring thermodynamic and structural stability of simulations, we analyzed resulting conformational ensembles using cpptraj from AmberTools^33^. We analyzed B-factors of Cα atoms (derived from root mean squared fluctuations along the simulations) after a single alignment to all Cα atoms as a metric for global flexibility. Additionally, we extracted dihedral angles of the protein backbone and calculated dihedral entropies based on resulting distributions after employing parameter-free kernel density estimation^55^. Integration over probability densities yields a thermodynamic entropy depicting a metric for local intrinsic flexibility of the protein backbone^56^. Independent dihedral entropies over all three backbone torsion angles were summed to yield a total dihedral entropy per residue. All values are presented as arithmetic average over two dimer sub-units. Interface residues were defined as all residues with at least one atom closer than 3 Å to the adjacent sub-unit.

### NMR spectroscopy

The NMR data were acquired at 20 °C on a Bruker Avance DRX-500 spectrometer equipped with a triple-resonance probe and z-gradients. Proteins and ligands were prepared in 50 mM phosphate buffer pH 6.9. The ^15^N-labelled SAMp73 concentration was in all cases ~200 μM, NQO1 variants (as purified) were prepared at ~300 μM. When necessary, ligands were added to a final concentration of 3 mM (dicoumarol) or 8 mM (FAD or NADH). The 2D ^1^H-^15^N HSQC (heteronuclear single-quantum coherence) experiments^57^ were acquired in the following conditions: (i) isolated ^15^N-SAMp73; (ii) ^15^N- SAMp73 with NQO1 WT; (iii) ^15^N-SAMp73 with NQO1 WT + NADH; (iv) ^15^N-SAMp73 with Δ50-NQO1 WT; (v) ^15^N-SAMp73 with Δ50-NQO1 WT + FAD; (vi) ^15^N-SAMp73 with Δ50-NQO1 WT + FAD + NADH; (vii) ^15^N-SAMp73 with NQO1 P187S; (viii) ^15^N-SAMp73 with NQO1 P187S + FAD; (ix) ^15^N-SAMp73 with NQO1 P187S + FAD + NADH; and, (x) ^15^N-SAMp73 with NQO1 WT + dicoumarol (with stoichiometric amounts of dicoumarol). Samples containing Δ50-NQO1 led to precipitation during the experiments; experiments in the presence of dicoumarol also led to precipitation. Control experiments were acquired with ^15^N-SAMp73 with FAD and ^15^N-SAMp73 with NADH; no differences in either chemical shifts or signal broadening were observed when compared with the spectrum of isolated ^15^N-SAMp73 (BMRB number 4413)^58^.

Spectra were acquired in the phase sensitive mode. Frequency discrimination in the indirect dimensions was achieved by using the echo/antiecho-TPPI method. The spectra were acquired with 2 K complex points in the ^1^H dimension, 128 complex points in the ^15^N dimension, and 200 scans. The carrier of the ^1^H dimension was set at the water frequency, and that of ^15^N at 120 ppm. The spectral widths used were 10 and 35 ppm in the ^1^H and ^15^N dimensions, respectively. Water was suppressed with the WATERGATE sequence^59^. Data were zero-filled to double the number of original points in both dimensions, apodized with shifted squared sine-bell functions in the two dimensions and Fourier transformed with the program TopSpin 1.3 (Bruker). The assignment of the spectrum of SAMp73 under our conditions was carried out by comparison of the chemical shifts of the signals, with that previously published^58^.

To analyse the differences between the 2D ^1^H-^15^N HSQC spectra of isolated SAMp73 and of the complexes, the chemical shift perturbation (CSP) was used. CSP was calculated as: 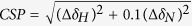, where Δ*δ*_*H*_ is the difference in chemical shift of the amide protons of isolated SAMp73 and that of SAMp73 in the corresponding complex; and Δ*δ*_*N*_ is the difference in chemical shift of ^15^N resonances of isolated SAMp73 and that of SAMp73 in the complex. Only variations in residues with CSP ≥ 0.01 ppm were considered significant. Broadening of signals was measured in comparison with the internal reference of Ala530. Changes in cross-peak intensity were considered significant when variations between the spectrum of isolated SAMp73 and that in the presence of NQO1 species were larger than 10%.

### Expression studies in a cell-free system

Expression in a rabbit reticulocyte cell-free system (TnT system, Promega) was performed at 30 °C for 30 min using ^35^S-Met and NOQ1 cDNA variants subcloned in pCIneo plasmids (Promega; 1 μg plasmid/reaction). Protein synthesis was stopped with 100 μg/ml cycloheximide, and kept at 30 °C for various chasing times. Aliquots were denatured in Laemmli’s buffer and analyzed by SDS-PAGE and fluorography. The autoradiograms were scanned in a Molecular Imager FX (Bio-Rad) and the software LabImager (Bio-Rad) was used to measure the band volume adjusted to background. All experiments were performed three independent times and data are presented as mean ± s.d.

## Additional Information

**How to cite this article:** Medina-Carmona, E. *et al*. Site-to-site interdomain communication may mediate different loss-of-function mechanisms in a cancer-associated NQO1 polymorphism. *Sci. Rep.* **7**, 44532; doi: 10.1038/srep44532 (2017).

**Publisher's note:** Springer Nature remains neutral with regard to jurisdictional claims in published maps and institutional affiliations.

## Supplementary Material

Supplementary Information

## Figures and Tables

**Table 1:** Thermal stability of NQO1 and Δ50 variants.

Variant	*T*_m_ (°C)		
	Apo	Holo	Holo + dic
WT	50.4	55.7	63.7
p.P187S	42.6	49.9	58.7
Δ50-WT	47.9	54.5	59.3
Δ50-p.P187S	39.5	48.7	56.3

The apparent melting temperatures (*T*_m_) are determined for proteins as apo-proteins, and in the presence of FAD or FAD + Dicoumarol.

**Table 2:** Enzyme kinetic parameters for holo NQO1 and Δ50 variants.

NQO1 variant	NADH		DCPIP	
	*k*_cat_ (s^−1^)	*K*_M_ (μM)	*k*_cat_ (s^−1^)	*K*_M_ (μM)
WT	177 ± 7	158 ± 21	199 ± 11	13 ± 2
p.P187S	124 ± 10	210 ± 48	160 ± 58	26 ± 20
Δ50-WT	6.6 ± 1.0	6422 ± 1668	1.3 ± 0.3	12 ± 8
Δ50-p.P187S	5.8 ± 0.3	9985 ± 797	0.74 ± 0.07	7 ± 3
