# Supplementary material for: Site-to-site interdomain communication may mediate different loss-of-function mechanisms in a cancer-associated NQO1 polymorphism
Source: Sci Rep. 2017 Mar 14;7:44532. doi: 10.1038/srep44532 (PMC5349528; doi:10.1038/srep44532)
Supplement: Supplementary Information [file srep44532-s1.pdf]

Site-to-site interdomain communication may mediate different loss-of-function mechanisms in a cancer-associated NQO1 polymorphism

Encarnación Medina-Carmona, Jose L. Neira, Eduardo Salido, Julian E. Fuchs, Rogelio Palomino-Morales, David J. Timson and Angel L. Pey.

Supplementary Information

Characterization of the oligomeric state of purified NQO1 variants

Size-exclusion chromatography (SEC).- To study the oligomeric state by SEC, samples were loaded onto a HiLoad 16/60 Superdex 200 pg (GE Healthcare) using 20 mM HEPES-NaOH, 200 mM NaCl pH 7.4 as mobile phase at 1.2 mL·min^{-1} flow rate. 2 mL of purified NQO1 samples at 4 μM (in monomer units) were injected. 2 mL of the following protein with known molecular weight were prepared at 0.2 mg·mL^{-1} and analyzed in the same way: *S. cerevisiae* alcohol dehydrogenase (150 kDa; SEC standard; from Sigma-Aldrich), bovine serum albumin (66 kDa; from Sigma-Aldrich), human phosphoglycerate kinase 1 (45 kDa, recombinantly expressed and purified by us; [1]), chicken egg white lysozyme (14.3 kDa, from Sigma-Aldrich). To determine the void (V_0=46 mL) and total (V_t=115 mL) volumes of the column, solutions of Blue dextran and pyridoxal 5´-phosphate were used (both from Sigma-Aldrich).

Dynamic light scattering (DLS).- To study the oligomeric state by DLS, NQO1 samples diluted in 20 mM HEPES-NaOH, 200 mM NaCl pH 7.4 at 9, 3 or 1 μM (in monomer units). DLS experiments were performed in a DynaPro MSX instrument (Wyatt) using 1.5 mm path length cuvettes and analyzed using the software provided by the manufacturer. Three replicates of each sample were prepared and analyzed.

Isothermal titration calorimetry (ITC).- To detect dimer dissociation, NQO1 enzymes (at 120-140 μM in monomer units) were loaded into the titrating syringe in 20 mM HEPES-NaOH, 200 mM NaCl pH 7.4, and the same buffer was applied to the calorimetric cell. Experiments were performed at 25°C in a ITC_{200} microcalorimeter

(Malvern). After an initial injection of 0.5 μL, 18 stepwise injections of 2 μL were performed. The final concentration of NQO1 enzymes in the cell was about 25 μM (in protein monomer).

Measurements of T_2 by NMR spectroscopy.- Measurements of T_2 (transverse relaxation time) provide a convenient method for determining the molecular mass of a macromolecule, since the correlation time of the molecule, τ_c, is approximately equal to $1/(5T_2)$ [2]. However, the measured values can be affected by the local, particular movements of the chosen nucleus, and then the T_2-value can be very small. We measured the T_2 for NQO1 WT and p.P187S, with the 1-1 echo sequence [3], and the calculation of the τ_c was carried out as previously described [2]. Experiments were carried out at 20 ºC, in phosphate buffer (50 mM, pH 7.5). Protein concentrations (in monomer units) were 20 μM. Additional information on equipment and conditions used can be found in the methods section of the main text.

Figure S1. Structural overlay between crystal structures of p.P187S full-length (PDB 4CF6) and Δ50-p.P187S (PDB 4CET), with the similar conformation of the bound FAD highlighted. The calculated RMSD of the two structures is 0.41Å (using PyMol; [4]).

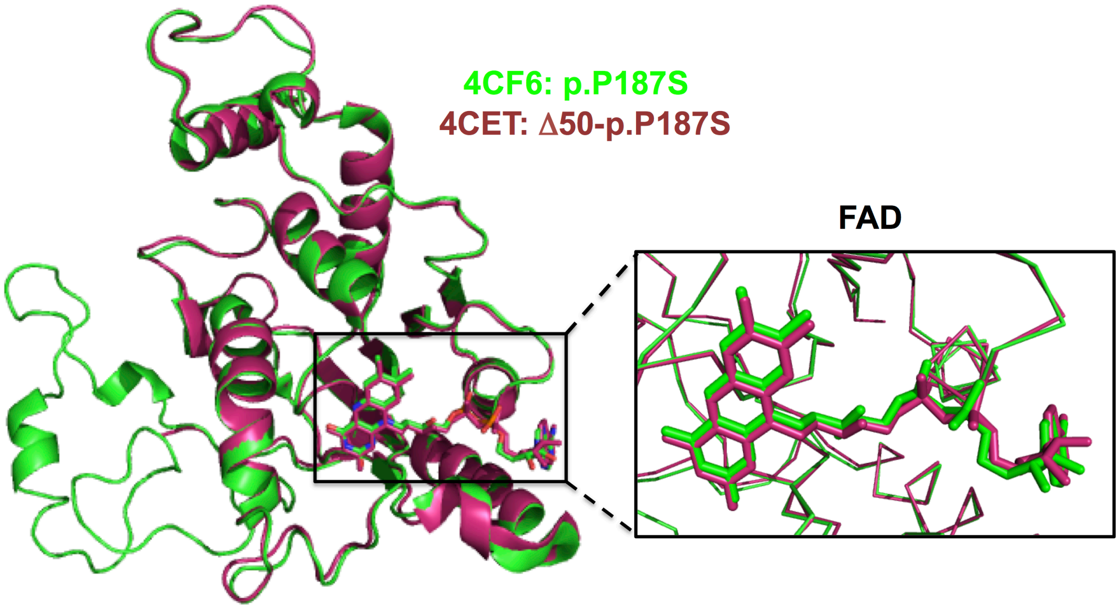

Figure S2. Oligomeric state of NQO1 variants as purified. A) SEC analyses of NQO1 proteins injected at 4 μM (in monomer unit), indicating the elution volume of proteins with known molecular weights; Note that during the chromatographic analyses, protein samples are diluted by ~5-fold (the peak width is of ~10 mL), and thus the real operational protein concentration can be considered as ~0.8 μM; B) DLS analyses (top to bottom panels show: hydrodynamic radius, apparent molecular weight and polydyspersity indexes; data are mean±s.d. from three independent samples). C) Dissociation studies by ITC, showing little or no thermal effect upon dilution of NQO1 dimers (120-140 μM in protein monomer) into the cell compared to the injection of buffer.

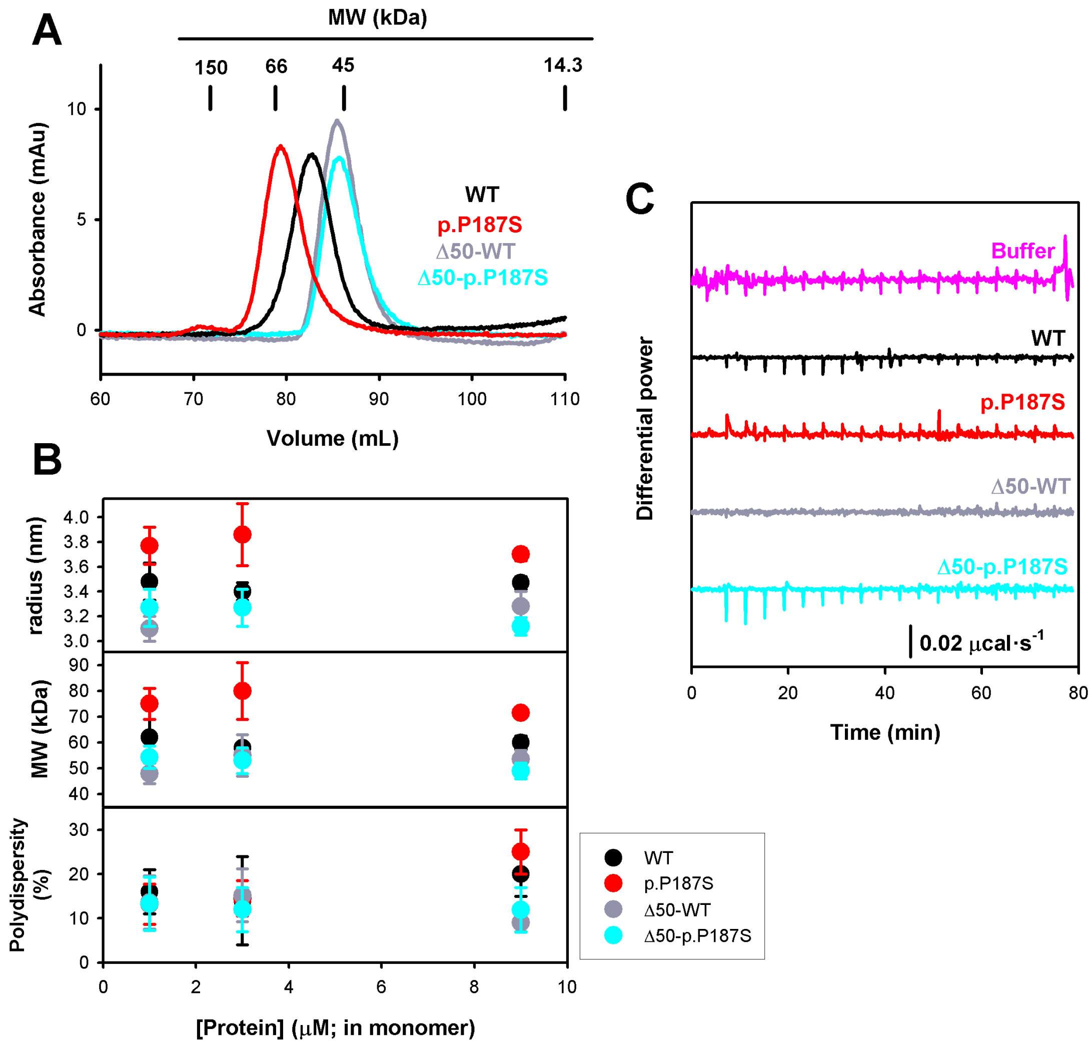

Figure S3. Proteolysis of Δ50 variants by thermolysin. Upper panel) Pictures of representative SDS-PAGE gels with two different brightness/contrast; Lower panel) Peptide fingerprinting of Δ50-WT selected proteolysis products by MALDI-TOF/TOF.

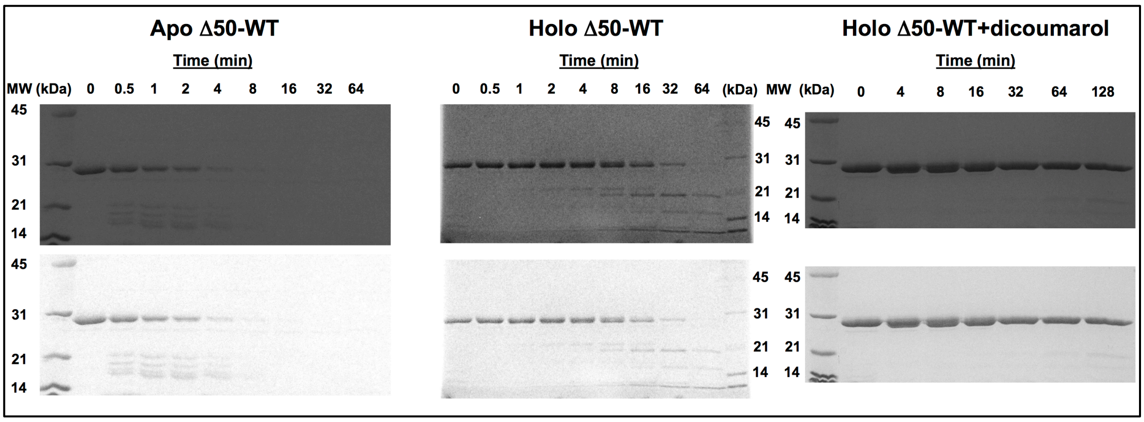


```
SAMPLE #1: MW: 25880 Da (HPLC/ESI-MS)

   -10  ↓      1                     21                    41
MAHHHHHHVD DDDKMVGRRA LIVLAHSERT SFNYAMKEAA AAALKKKGWE VVESDLYAMN

               61                    81                   101
FNPIISRKDI TGKLKDPANF QYPAESVLAY KEGHLSPDIV AEQKKLEAAD LVIFQFPLQW

              121                   141                   161
FGVPAILKGW FERVFIGEFA YTYAAMYDKG PFRSKKAVLS ITTGGSGSMY SLQGIHGDMN

              181                   201                ↓
VILWPIQSGI LHFCGFQVLE PQLTYSIGHT PADARIQILE GWKKRLENIW DETPLYFAPS

Peptide 1: VDDDDKMVGRR (from -5 to 5)
Péptide 2: ALIVLAHSER (from 6 to 15)
Peptide 3: TSFNYAMK (from 16 to 23)
Peptide 4: KGWEVVESDLYAMNFNPIIS (from 33 to 52)
Peptide 5: LKDPANFQYPAESVLAYK (from 60 to 77)
Peptide 6: AAMYDKGPFR (from 130 to 139)
Peptide 7: KRLENIWDETPLY (from 210 to 222)

Cleavage at ↓ provides a species with 25697.5 Da (from -5 to 221).


SAMPLE #2: MW: 18190 or 17500 Da (HPLC/ESI-MS)

   -10  ↓      1                     21                    41
MAHHHHHHVD DDDKMVGRRA LIVLAHSERT SFNYAMKEAA AAALKKKGWE VVESDLYAMN

               61                    81                   101
FNPIISRKDI TGKLKDPANF QYPAESVLAY KEGHLSPDIV AEQKKLEAAD LVIFQFPLQW

              121                   141↓    ↓          ↓  161
FGVPAILKGW FERVFIGEFA YTYAAMYDKG PFRSKKAVLS ITTGGSGSMY SLQGIHGDMN

              181                   201                   221
VILWPIQSGI LHFCGFQVLE PQLTYSIGHT PADARIQILE GWKKRLENIW DETPLYFAPS

Péptido 1: VDDDDKMVGRR (from -5 to 5)
Péptido 2: TSFNYAMK (from 16 to 23)
Péptido 3: KKGWEVVESDLYAMNFNPIISR (from 32 to 53)
Péptido 4: LKDPANFQYPAESVLAYK (60-77)
Péptido 5: EGHLSPDIVAEQK (78-90)
Péptido 6: VFIGEFAYTYAAMYDK (120-135)
Péptido 7: AAMYDKGPFR (from 130 to 139)

Cleavage at ↓ provides a species with 16872 Da (from -5 to 143), 17172
Da (from -5 to 146) or 18222 Da (from -5 to 157).
```

Figure S4. ITC binding isotherms for the interaction of NQO1 enzymes with FAD (A, apo-proteins) and dicoumarol (B and C, holo-proteins). Lines are best-fits to a model for a single type of identical and independent sites, and thus, imply the absence of cooperative effects between a type of binding sites.

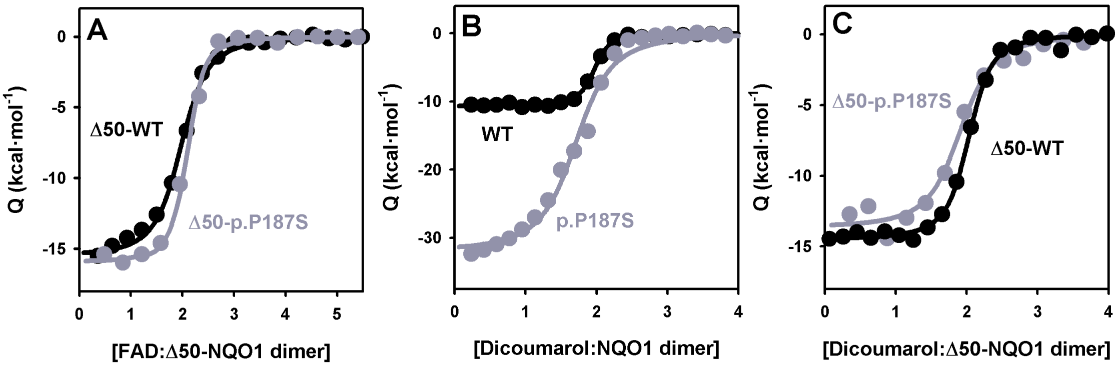

Figure S5. Dynamic effects (dihedral entropies) of truncation of NQO1 on its C-terminal domain and ligand binding investigated by MD simulations A and B) Effects on the global (A) and dimer interface (B) dynamics of full-length and truncated NQO1 variants in different ligation states (apo, holo, and holo+dicoumarol); C-D) Difference between C-terminal truncated and full length dynamics for WT (C) and p.P187S (D) as apo-proteins (apo), holo-proteins (holo) and holo-proteins with dicoumarol bound (dic); E-G) Difference between p.P187S and WT as full-length and C-terminal truncated proteins in different ligation states (E, apo; F, holo; G, holo+dicoumarol).

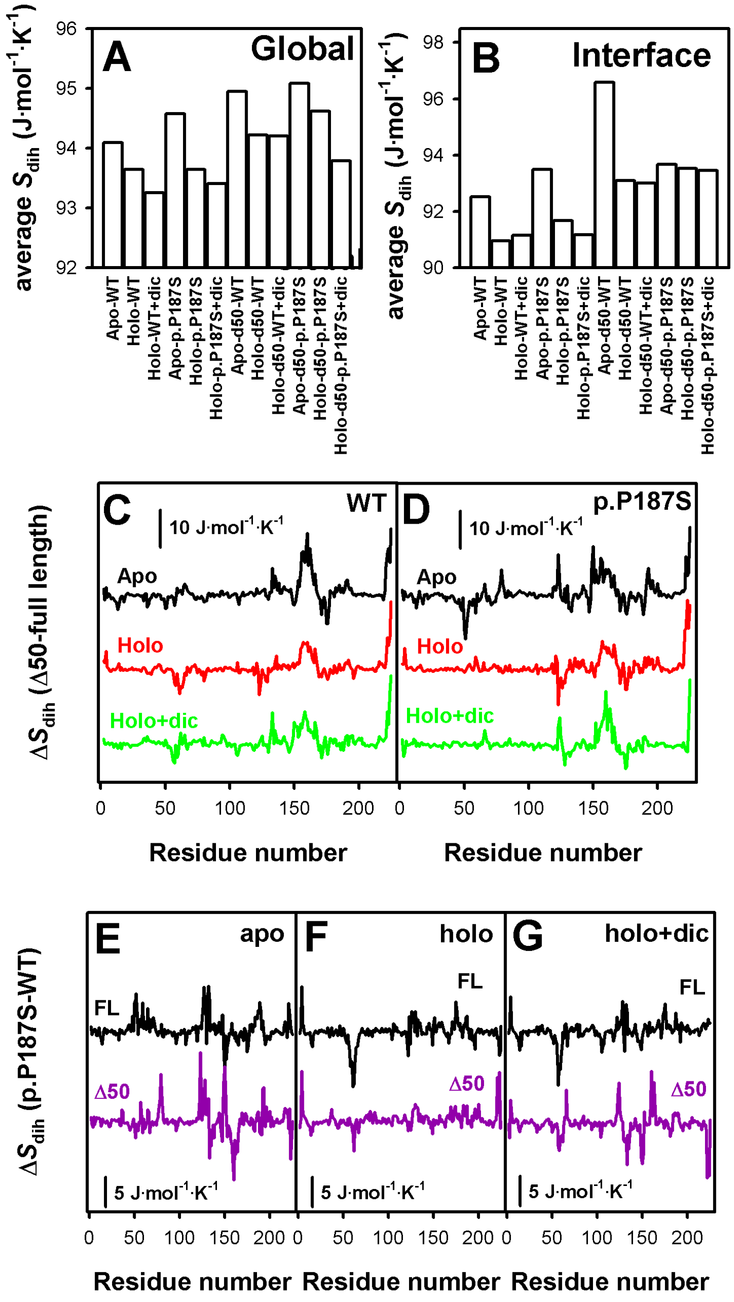

Figure S6. Fluorescence titration of NQO1 WT with SAMp73. NQO1 (4.2 μM) was incubated with different concentrations of SAMp73 (0-8 μM) at 25°C and fluorescence intensity was registered (excitation at 280 nm; emission at 350 nm). The intensity corresponding to free SAMp73 (without NQO1) was determined in independent measurements and consequently subtracted.

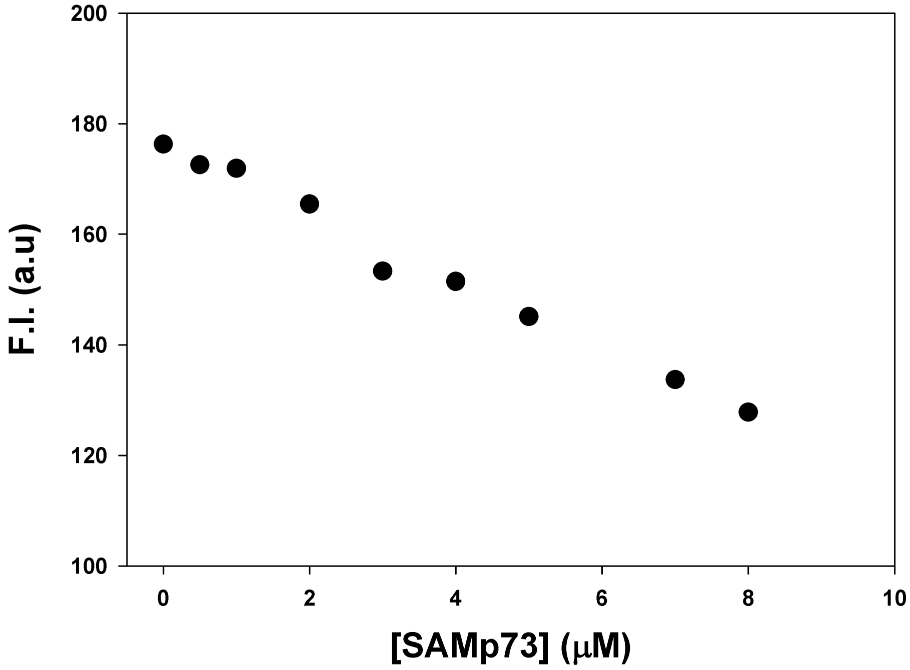

Figure S7. Up-field shifted region of the ^{15}N-^{1}H HSQC spectra of SAMp73 under different conditions. The regions of the glycines cross-peaks of isolated SAMp73 (black), with NQO1 WT (blue), and with NQO1 WT+NADH (orange) are shown. The glycines are numbered according to the numbering of the whole p73. Experiments were acquired at pH 6.9 (50 mM phosphate) and 20 ºC.

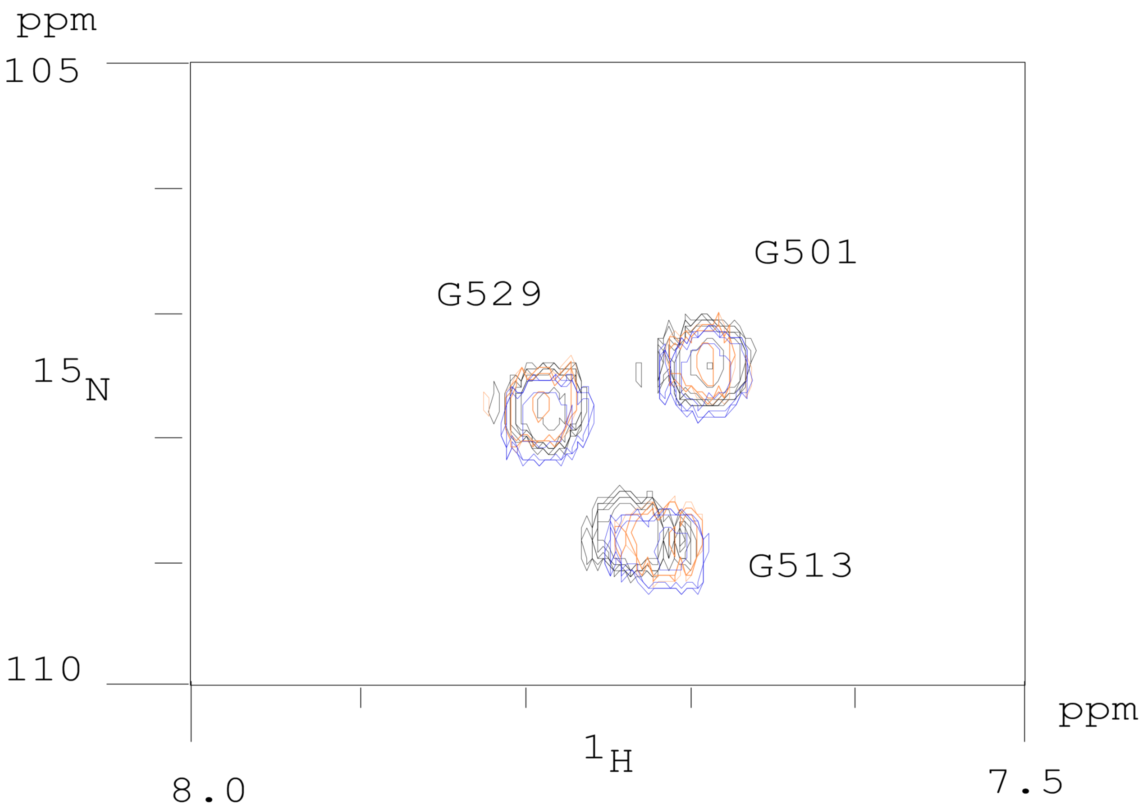

Table S1. Proteolysis products of Δ50-WT by HPLC-ESI/MS. Reaction times are indicated. Thermolysin was used at 100 nM. Temperature was 25 ^{o}C.

**Conditions**		
**Apo, 1 min**	**Holo, 16 min**	**Holo+dicoumarol, 4 h**
26760	26760	25880
19045	18190	18190
18010	17500	17000
17165	15360	15305
16665		

Table S2. Residues of SAMp73 whose chemical shifts and/or broadening of cross-peaks were affected by the presence of NQO1 species.

NQO1	Condition	Chemical shift changes[a]	Broadening changes
WT[b]	As purified	F509 (0.02), L514 (0.02) (F496)[c], G513 (0.03), I533 (0.01)	D490, S492, L493 (0.02)[d], E535, Q536, I541, L545 (I517)
	+NADH	G513 (0.03)	D490, L493 (0.02)[d], E535, I541, W542
Δ50-WT[b]	As purified		Y487 (I541), L493 (0.02)[d], N504 (M539), G513, E535
	+FAD		Y487 (I541), L493 (0.02)[d], N504 (M539), G513, E535
	+FAD+NADH		All cross-peaks became broader
p.P187S[b]	As purified	G513 (0.03), Q536 (0.01) (R543)	Y487 (I541), L493 (0.02)[d], N504 (M539), E535, L545
	+FAD	Y508 (0.02), G513 (0.03)	Y487 (I541), L493 (0.02)[d], N504 (M539), E535, L545
	+FAD+NADH	Y508 (0.02), G513 (0.02)	Y487 (I541), L493 (0.02)[d], N504 (M539), E535

[a] Only residues with CSP ≥ 0.01 ppm are reported (the values of the CSP are reported beside each residue). Assignments of the isolated SAMp73 were taken from BMRB (BMRB accession number 4413).

[b] WT, Δ50-WT and p.P187S as purified contained high, low and negligible levels of FAD, respectively.

[c] Residues within parenthesis indicate overlapping with the indicated preceding residue.

[d] Residues which showed also changes in CSP ≥ 0.01 ppm.

References

1 Pey, A. L., Mesa-Torres, N., Chiarelli, L. R. & Valentini, G. Structural and Energetic Basis of Protein Kinetic Destabilization in Human Phosphoglycerate Kinase 1 Deficiency. *Biochemistry* **52**, 1160-1170 (2013).

2 Anglister, J., Grzesiek, S., Ren, H., Klee, C. B. & Bax, A. Isotope-edited multidimensional NMR of calcineurin B in the presence of the non-deuterated detergent CHAPS. *J Biomol NMR* **3**, 121-126 (1993).

3 Sklenar, V. & Bax, A. Spin echo water suppression for the generation of pure-phase two-dimensional NMR spectra. *J. Magn. Reson.* **74**, 469-479 (1987).

4 DeLano, W.L. (2002) The PyMOL Molecular Graphics System. DeLano Scientific LLC, Palo Alto, CA, USA. http://www.pymol.org.
